# Gut fungal dysbiosis correlates with reduced efficacy of fecal microbiota transplantation in *Clostridium difficile* infection

**DOI:** 10.1038/s41467-018-06103-6

**Published:** 2018-09-10

**Authors:** Tao Zuo, Sunny H. Wong, Chun Pan Cheung, Kelvin Lam, Rashid Lui, Kitty Cheung, Fen Zhang, Whitney Tang, Jessica Y. L. Ching, Justin C. Y. Wu, Paul K. S. Chan, Joseph J. Y. Sung, Jun Yu, Francis K. L. Chan, Siew C. Ng

**Affiliations:** 1 Department of Medicine and Therapeutics, The Chinese University of Hong Kong, Hong Kong, China; 2 Institute of Digestive Disease, State Key Laboratory of Digestive Diseases, LKS Institute of Health Science, The Chinese University of Hong Kong, Hong Kong, China; 3 Center for Gut Microbiota Research, The Chinese University of Hong Kong, Hong Kong, China; 4 Shenzhen Research Institute, The Chinese University of Hong Kong, Shenzhen, China; 5 Department of Microbiology, The Chinese University of Hong Kong, Hong Kong, China

## Abstract

Fecal microbiota transplantation (FMT) is effective in treating recurrent *Clostridium difficile* infection (CDI). Bacterial colonization in recipients after FMT has been studied, but little is known about the role of the gut fungal community, or mycobiota. Here, we show evidence of gut fungal dysbiosis in CDI, and that donor-derived fungal colonization in recipients is associated with FMT response. CDI is accompanied by over-representation of *Candida albicans* and decreased fungal diversity, richness, and evenness. Cure after FMT is associated with increased colonization of donor-derived fungal taxa in recipients. Recipients of successful FMT (“responders”) display, after FMT, a high relative abundance of *Saccharomyces* and *Aspergillus*, whereas “nonresponders” and individuals treated with antibiotics display a dominant presence of *Candida*. High abundance of *C. albicans* in donor stool also correlates with reduced FMT efficacy. Furthermore, *C. albicans* reduces FMT efficacy in a mouse model of CDI, while antifungal treatment reestablishes its efficacy, supporting a potential causal relationship between gut fungal dysbiosis and FMT outcome.

## Abstract (web summary)

Fecal microbiota transplantation (FMT) is effective in treating recurrent *Clostridium difficile* infection (CDI). Here, the authors show that the composition of the gut fungal microbiota of donors and recipients, and especially the abundance of *Candida*, correlates with FMT outcome in CDI patients.

## Introduction

The past decade has witnessed an increasing use of fecal microbiota transplantation (FMT) as a promising treatment option for several diseases^1–3^, yet success rates are variable with a cure rate of 85–90% in recurrent *Clostridium difficile* infection (CDI)^3–5^, and a response rate of 30–50% in inflammatory bowel disease (IBD)^6–8^. Such variations may be related to disease traits, recipient factors or donor characteristics. The mechanisms underlying a successful FMT and its relationship with gut microbial profiles in donor–recipient pairs remain elusive. To date, the efficacy of FMT has been mostly ascribed to the restoration of a normal bacterial microbiota composition and function, and a sustained coexistence of donor and recipient bacterial strains^9–12^. Recently, bacteriophages have been shown to be altered in CDI after FMT and these changes were associated with treatment outcome^13–15^. The human gastrointestinal tract is also colonized by a large population of fungi, collectively referred to as the mycobiota, which play an important role in human health^16,17^. Our gut mycobiota contributes to normal human physiology and in some cases can recapitulate the benefit of intestinal bacteria via regulating host immunity and maintaining intestinal homeostasis^16–18^. Whether donor-derived mycobiota can colonize a recipient host, the fate of donor and recipient mycobiota after FMT and their relationship with treatment outcomes are unknown.

Here, we study the gut mycobiota of FMT-treated subjects with CDI to explore the effects of FMT on the gut mycobiome in association with treatment outcome. We show enteric mycobiota dysbiosis in CDI subjects, and that cure after FMT is more frequently observed when donor-derived fungal taxa predominate in the recipient mycobiota. The gut mycobiome of FMT nonresponders and antibiotic-treated CDI subjects displays a dominant presence of *Candida and Candida albicans* after treatment. Hence, *C. albicans* abundance is associated with unfavorable FMT outcome in humans. Furthermore, we show that *C. albicans* compromises FMT efficacy in a mouse model of CDI.

## Results

### Gut fungal dysbiosis in CDI

**Fig. 1:**
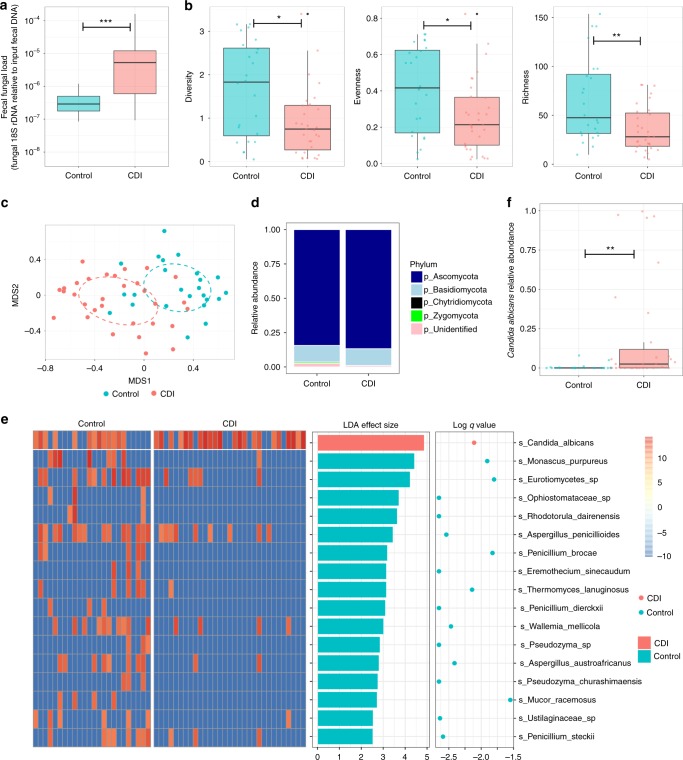
Fungal alterations in CDI. **a** Comparison of the total fungal load in the feces of controls and CDI subjects. Statistical significance was determined by Mann–Whitney test, ^***^P < 0.001. **b** Comparison of the fecal mycobiota based on Shannon diversity, evenness, Chao1 richness in controls and CDI subjects. The dots indicate individual values of the studied subjects. Statistical significance was determined by Mann–Whitney test, *P < 0.05, ^**^P < 0.01. **c** Fungal community structure difference between controls and CDI by NMDS (nonmetric multidimensional scaling) plot based upon Bray–Curtis dissimilarities. **d** Comparison of the fecal mycobiota composition between controls and CDI subjects at the phylum level. **e** Differentially enriched fungal species between controls and CDI. Statistical significance was determined by LefSe analysis with FDR correction (only those species with *q* values < 0.05 and LDA effect size > 2 are shown). Heatmap of the presence of these differential fungal species is shown in relative abundance intensity. LDA effect size, log_10_ transformed *q* value (FDR-adjusted *p* value) and species annotation are shown. Green bars and dots indicate species enriched in controls, while red bars and dots indicate species enriched in CDI. **f** Comparison of the relative abundance of fecal *C. albicans* in controls and CDI subjects. Statistical significance was determined by Mann–Whitney test, ^**^P < 0.01. For box plots, the boxes extend from the first to third quartile (25th to 75th percentile), with the median depicted by a vertical line

We compared the fecal mycobiomes of 31 CDI subjects and 24 healthy controls. The total fecal fungal load was higher in CDI than in controls [Mann–Whitney test, *p* = 0.0004, log_10_ transformed effect size 1.32 (95% CI: 0.62–1.97), Fig. 1a]. However, there was a decrease in fungal diversity, evenness, and richness in CDI compared with controls (Mann–Whitney test, *p* = 0.0120, 0.0309, and 0.0043, respectively, Fig. 1b). The fungal communities of CDI subjects were separated from those of healthy controls at the operational taxonomic unit (OTU) level (based on Bray–Curtis dissimilarities, adonis test *p* = 0.003, Fig. 1c). At the phylum level, *Ascomycota* was expanded in CDI compared with controls (Mann–Whitney test, *p* = 0.0083, Fig. 1d). At the species level, 17 fungal species were found to be differentially present between CDI and controls (LefSe analysis with false discovery rate (FDR) adjusted *q* < 0.05, Fig. 1e and Supplementary Data 1). Among these species, only *C. albicans* was significantly enriched in CDI (Fig. 1e, f, Mann–Whitney test, *p* = 0.0080), whereas 16 other species were enriched in controls. We then used quantitative PCR to determine the absolute abundance of *C. albicans* in feces and confirmed the over-presentation of *C. albicans* in CDI [Mann–Whitney test, *p* < 0.0001, log_10_ transformed effect size 1.53 (95% CI: 0.90–2.11), Supplementary Figure 1a].

Antibiotic use has been shown to be a major contributor to the development of CDI by decreasing bacterial colonization resistance^19,20^. Antibiotic use has also been reported to promote the growth of *C. albicans*^21,22^. We further assessed the effect of antibiotics on *C. albicans* levels in CDI. We collected stool samples from consecutive new CDI patients, including 12 CDI patients with antibiotics exposure, 12 CDI patients with no antibiotics exposure at inclusion, and 17 healthy controls. We found significantly higher levels of fecal *C. albicans* in CDI subjects exposed to antibiotics at inclusion, compared with controls (Supplementary Figure 1b, Mann–Whitney test, *p* = 0.0131). *C. albicans* levels were also higher in CDI subjects not exposed to antibiotics at inclusion when compared with controls (Supplementary Figure 1b, Mann–Whitney test, *p* = 0.0469). These data suggest that antibiotics may not be the only contributor to increased levels of *C. albicans* and that *C. difficile* infection *per se* is also associated with *C. albicans* bloom.

In line with the observation at the species level, more taxa were enriched in controls than in CDI, as determined by LefSe analysis (9 versus 1 at the order level, 15 versus 2 at the family level, and 16 versus 1 at the genus level, Supplementary Data 2). Altogether, these data indicate dysbiosis of the enteric mycobiota in patients with CDI. MaAsLin analysis identified no significant correlations between discriminative fungal taxa and subject metadata (age, gender, household, CDI severity, and underlying IBD).

### FMT alters the gut mycobiota in relation to treatment response

We then explored whether FMT leads to colonization of donor-derived fungi and its association with treatment efficacy. Changes in the gut mycobiomes of recipients after FMT were monitored at multiple time-points in 16 CDI subjects, using pre-FMT samples of each donor–recipient pair as baseline for FMT (Supplementary Figure 2). Among 16 CDI subjects treated with FMT, 9 remained symptom-free with a negative stool *C. difficile* toxin at the last follow-up (termed “responders”, FMT1–FMT9), while seven developed recurrence of CDI (termed “nonresponders”, FMT10–FMT16) (Supplementary Data 3). We next investigated whether donor-derived fungi and bacteria in recipients were associated with FMT outcomes. Subjects who responded to FMT demonstrated a larger proportion of fungal and bacterial OTUs that were transferred and predominated in the fecal microbiota of recipients after FMT, compared to those who did not respond (Mann–Whitney test, *p* = 0.0068 and 0.0164, respectively for comparisons of donor-derived fungal and bacterial OTU ratios in recipients, Supplementary Figure 3a, b).

**Fig. 2:**
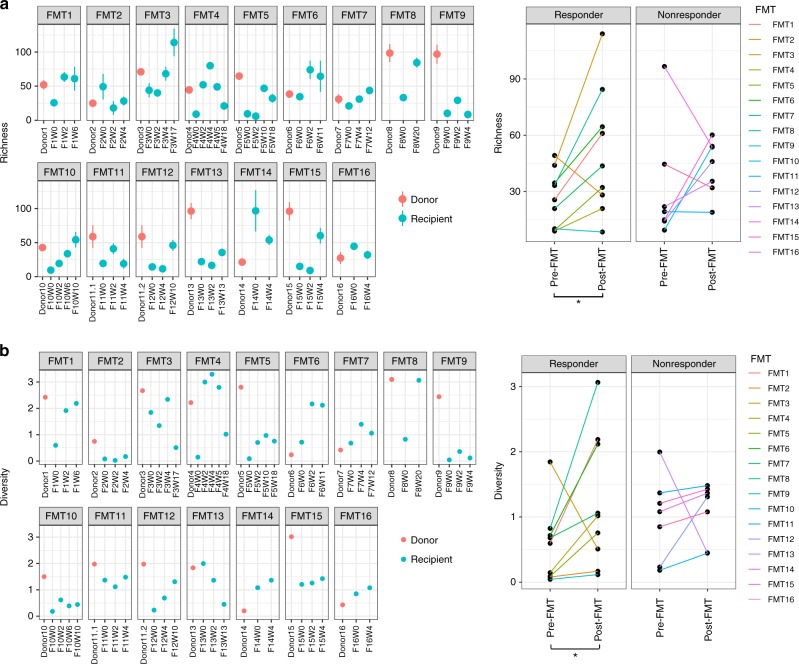
Post-FMT alterations in the enteric mycobiota richness and diversity of CDI recipients in association with FMT response. Fecal fungal richness (**a**) and diversity (**b**) alterations in FMT recipients over the course of longitudinal follow-up and in their corresponding donors at baseline. Comparison of the fungal richness and diversity of pre-FMT samples and post-FMT samples collected at the last follow-up are shown in FMT responders and FMT nonresponders, respectively. Statistical significance was determined by paired Wilcoxon signed rank test, ^*^p < 0.05. “F” indicates FMT-treated subject. “W” indicates weeks post-treatment

**Fig. 3:**
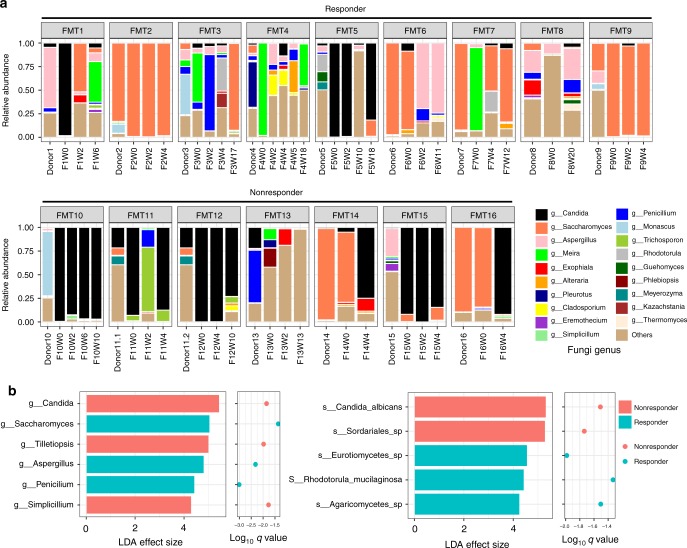
Post-FMT alterations in the enteric mycobiota composition of CDI recipients in association with FMT response. **a** Alterations in the fecal fungal composition at the genus level in CDI recipients after FMT at different time-points up to the last follow-up. **b** Differentially enriched fungal taxa across all post-FMT fecal samples of FMT responders versus nonresponders at the genus and species levels. Statistical significance level was determined by LefSe analysis with FDR correction (only those taxa with *q* values < 0.05 and LDA effect size > 2 are shown) and adjustment for subject number. Green bars and dots indicate taxa enriched in FMT responders, while red bar and dot indicate taxa enriched in FMT nonresponders

During follow-up, FMT responders showed an increase in fungal richness and diversity after FMT (Wilcoxon matched-pairs singed rank test, *p* = 0.0273 and *p* = 0.0474, respectively, Fig. 2a, b). There were profound differences in the gut mycobiota configurations across different donor–recipient pairs following FMT. The relative abundance of the genus *Candida* was higher in FMT nonresponders compared with responders across serial post-FMT fecal samples (Fig. 3a, b). Discriminative analysis identified disparately presented fungal taxa between post-FMT samples of FMT responders and nonresponders, at the genus and species level (Fig. 3b). Post-FMT, the genera *Saccharomyces*, *Aspergillus*, and *Penicillum* were relatively more abundant in FMT responders than in nonresponders. At the species level, *C. albicans* was the most prominent species enriched in nonresponders after FMT.

We also investigated alterations in the bacterial microbiota both at baseline, between CDI and controls, and after FMT in CDI recipients. Bacterial diversity, evenness and richness at baseline were lower in CDI subjects than controls (Mann–Whitney test, all *p* < 0.01, Supplementary Figure 4a). After FMT there was a significant increase in bacterial richness (Wilcoxon matched-pairs singed rank test, *p* = 0.019) and a marginally significant increase in bacterial diversity (Wilcoxon matched-pairs singed rank test, *p* = 0.098) in FMT responders (Supplementary Figure 4b, c). We explored the composition of the bacterial microbiota after FMT in relation to FMT outcomes, at various taxonomic levels (Supplementary Figure 5). The Actinobacteria and Bacteroidetes phyla, the *Lachnospiraceae*, *Clostridiaceae*, and *Ruminococcaceae* families, and the *Clostridium*, *Blautia*, and *Faecalibacterium* genera were enriched in FMT responders in comparison with nonresponders after FMT. However, bacteria from the phylum Proteobacteria were more abundant in FMT nonresponders relative to FMT responders. FMT responders displayed bacterial abundances resembling that of the donor, whereas FMT nonresponders showed inadequate relative abundances of donor-enriched bacteria at the last follow-up after FMT (Supplementary Figure 5c). In recipients FMT12 and FMT16, bacterial configurations at the last follow-up after FMT were similar to that of healthy individuals, but their gut mycobiota configurations differed significantly from that of healthy individuals (Fig. 3a). Taken together, these data suggest that the final proportion of donor-derived fungal and bacterial taxa and alterations of the fecal fungal composition in the recipient post-FMT are associated with FMT outcome.

### *C. albicans* is associated with poor FMT outcome

**Fig. 4:**
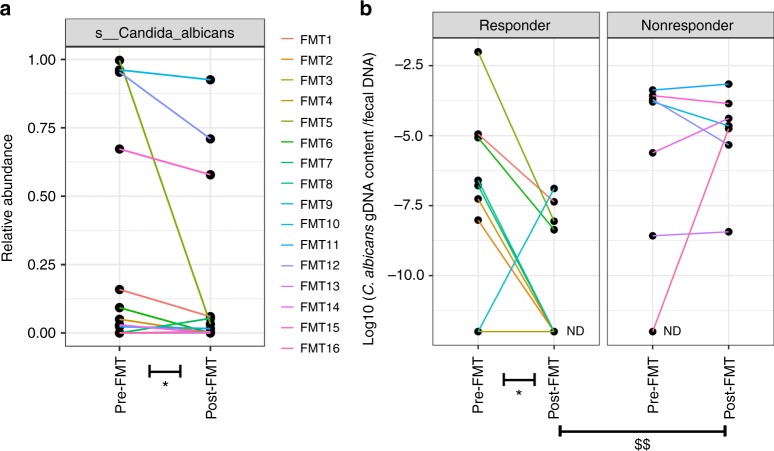
The presence of *C. albicans* is linked to FMT outcomes in CDI. **a** Alterations in the relative abundance of fecal *C. albicans* before and after FMT at the last follow-up in FMT recipients, assessed by ITS sequencing. The relative abundance of fecal *C. albicans* was calculated as the proportion of sequences taxonomically binned as *C. albicans* relative to the sequences classified as fungi. Statistical significance was determined by paired Wilcoxon signed rank test, ^*^p < 0.05. **b** The absolute fecal *C. albicans* levels before and after FMT at the last follow-up in FMT recipients, assessed by quantitative PCR. For those FMT nonresponders, the last follow-up time-point was a time prior to CDI recurrence after FMT. Comparison of the fecal *C. albicans* levels between pre-FMT samples and post-FMT samples was performed by paired Wilcoxon signed rank test, ^*^p < 0.05. Comparison of the fecal *C. albicans* levels between the post-FMT samples of FMT responders and FMT nonresponders was performed by Mann–Whitney test, ^$$^*p* < 0.01. ND denotes no detectable *C. albicans* in the feces

The relative abundance of *C. albicans* within the fecal fungal community was decreased after FMT (Wilcoxon matched-pairs singed rank test, *p* = 0.0458, Fig. 4a). Recipients with an initial high relative abundance of *C. albicans* before FMT and who continued to have a high relative abundance of *C. albicans* > 10% after FMT experienced disease recurrence after FMT (Fig. 4a). In accordance, the absolute abundance of *C. albicans* (in fecal input DNA) was markedly decreased after FMT in FMT responder group (Wilcoxon matched-pairs singed rank test, *p* = 0.0391, Fig. 4b). Interestingly, FMT nonresponders exhibited higher post-FMT fecal *C. albicans* levels in absolute abundance than FMT responders [Mann–Whitney test, *p* = 0.0018, log_10_ transformed effect size 3.05 (95% CI: 1.48–4.29), Fig. 4b]. Discriminative analysis via LefSe on the fecal mycobiomes between donors corresponding to FMT responders and nonresponders discerned *C. albicans* as the only significantly differential species enriched in FMT nonresponders’ donor stool (least discriminant analysis (LDA) effect size 2.25, FDR-adjusted *q* = 0.029). Accordingly, LefSe analysis on the fecal bacteriomes of donors at the genus level identified *Escherichia* and *Proteus* as the differentially enriched genera in FMT responders’ donor stool and in FMT nonresponders’ donor stool respectively (LDA effect size 2.58 and 2.35, FDR-adjusted *q* = 0.017 and 0.006, respectively). These data demonstrated that the abundance of *C. albicans* both in post-FMT recipient feces and in donor feces correlate with FMT outcome.

We subsequently screened and selected two new donors with undetectable fecal *C. albicans*, as determined by qPCR, and transplanted their fecal preparations into five new CDI recipients (Supplementary Figure 6a). After FMT, four CDI recipients showed low fecal *C. albicans* levels (Supplementary Figure 6b). One recipient who continued to show high level of *C. albicans* after FMT (Case 1) had early recurrence of CDI (Supplementary Figure 6).

### Antibiotic treatment leads to gut mycobiota alterations distinct from FMT treatment

We also assessed the effect of antibiotics on the gut mycobiota across longitudinal time-points in eight CDI subjects treated with vancomycin (STD treatment, Supplementary Figure 2, Supplementary Data 3). Five of the eight subjects remained symptom-free with negative stool *C. difficile* toxins at last follow-up (termed “responders”, STD1–STD5), while three developed recurrence of CDI (termed “nonresponders”, STD6–STD8). Unlike FMT, vancomycin induced inconsistent alterations in the fungal richness and diversity during longitudinal follow-up (Supplementary Figure 7a, b). FMT and vancomycin led to an increase in the gut fungal diversity in 81.3% (13 out of 16) and 37.5% (3 out of 8) of CDI subjects respectively (chi-square test *p* = 0.032, Supplementary Figure 7c), and an increase in the gut fungal richness in 68.8% (11 out of 16) and 37.5% (3 out of 8) of CDI subjects respectively (Supplementary Figure 7c). FMT responders showed a higher fold-change post-FMT in mycobiota richness when compared to STD responders (Student’s *t* test, *p* = 0.0231), while differences in mycobiota diversity between FMT responders and STD responders were not statistically significant (Supplementary Figure 7d, e). There was no significant difference in the bacterial richness or diversity between STD responders and nonresponders after vancomycin treatment, although vancomycin treatment resulted in a significant increase in bacterial diversity in STD responders (Supplementary Figure 8, Wilcoxon matched-pairs singed rank test, *p* = 0.0198). Collectively, these data suggest that FMT may induce alterations in the gut mycobiota more effectively than antibiotics.

We further performed taxonomical analysis to study the effect of antibiotics on the fungal community and to determine differences between FMT and antibiotics treatment in modulating the gut mycobiota. After vancomycin treatment, fungal compositions exhibited similar configurations across nearly all recipients during follow-up with a marked increase of the genus *Candida* (Supplementary Figure 9a). Mice also demonstrated augmented fecal *Candida* levels post vancomycin treatment (Supplementary Figure 9b, Wilcoxon matched-pairs singed rank test, *p* = 0.0156). There was no significant difference in the relative or absolute abundance of *C. albicans* between STD responders and nonresponders (Supplementary Figure 9c, d). Subject STD7, who showed a high post-STD relative abundance of *C. albicans* (>10%) and high absolute abundance in feces, developed CDI recurrence after vancomycin treatment, further supporting a correlation between *C. albicans* abundance and CDI outcome.

To define differentially enriched fungal taxa with respect to FMT and vancomycin treatment, we implemented LefSe analysis across all the follow-up samples of subjects corresponding to these two regimes. FMT treatment enriched the genera *Saccharomyces* and *Cryptococcus* in post-FMT samples, whereas vancomycin disparately enriched a panel of fungal genera after treatment, which included *Candida*, *Talaromyces*, *Erythrobasidium*, *Periconia*, *Stemphylium*, and* Ganoderma* (Supplementary Figure 10). At the family level, FMT was associated with enrichment of *Saccharomycetacea* and *Herpotrichiellaceae* (Supplementary Figure 10).

To characterize the ecological network of the gut mycobiota and bacterial microbiota, we evaluated the correlation of the α-diversity (diversity, evenness, and richness) of the fungal community with that of the bacterial community. Among the post-treatment samples of FMT responders, significant positive correlations were found between fungal diversity and bacterial diversity, and between fungal richness and bacterial diversity, evenness, and richness (Spearman’s correlation, permutation test, *p* < 0.05, Supplementary Figure 11). In the post-treatment samples of FMT nonresponders and STD responders, the α-diversity correlation between bacterial and fungal communities displayed a depletion of correlations between fungal richness and other bacterial and fungal α diversity indexes. Correlations were completely abolished across the post-treatment samples of STD nonresponders.

### Gut fungal dysbiosis correlates with FMT efficacy in a CDI mouse model

**Fig. 5:**
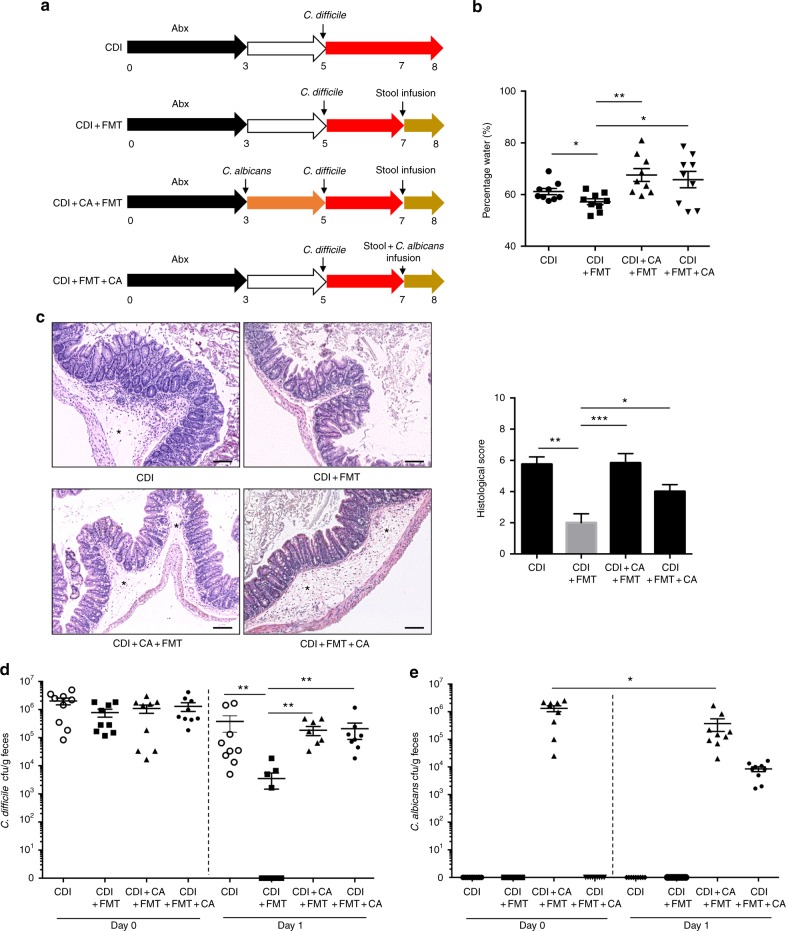
*C. albicans* compromises FMT efficacy in eradicating *C. difficile* infection in mice. **a** Schematic diagram of *C. albicans* administration and stool infusion (FMT) in a murine *C. difficile* infection (CDI) model. Antibiotic treatment was ceased before gavage of *C. albicans* (CA) and *C. difficile*. Number denotes day across the timeline of the experiment. **b** Diarrhea in mice on day 1 after FMT. Stool percentage water was expressed as mean ± s.e.m. Statistical significance represents comparisons by *t* test, ^*^p < 0.05, ^**^p < 0.01. **c** Representative H&E-stained cecum sections on day 1 after stool infusion (asterisk denotes edema). Scale bar, 100 µm. Histological scores were assessed and expressed as mean ± s.e.m. *n* = 6 mice per group. Statistical significance represents comparisons by *t* test, ^*^P < 0.05, ^**^p < 0.01, ^***^p < 0.001. **d** Enumeration of *C. difficile* in mouse feces on day 0 before FMT and day 1 post-FMT (*n* = 9 mice per group). Statistical significance represents comparisons of FMT-treated mice with *C. difficile* infection versus other groups by unpaired Mann–Whitney test. ^*^p < 0.05, ^**^p < 0.01. **e** Enumeration of *C. albicans* in mouse feces both on day 0 before FMT and on day 1 post-FMT (*n* = 9 mice per group). Statistical significance represents comparison between *C. albicans* load on day 0 before FMT and day 1 post-FMT, by paired Mann–Whitney test. ^*^p < 0.05. Dot graphs show means ± s.e.m, performed at least two times independently

Our results, showing *C. albicans* as the most prominent species associated with treatment failure of FMT in CDI, suggested a possible causal relationship. This assumption is further supported by reports whereby CDI recurrence was observed after antibiotics treatment^3,23^, as antibiotics contribute to the expansion of *Candida*. To explore a potential causal relationship between *C. albicans* and an unfavorable FMT response, we assessed the efficacy of FMT in eliminating *C. difficile* using a *C. difficile*-induced diarrhea murine model in three groups of mice with a control group of CDI mice without FMT treatment (CDI): (i) mice infused with human stool preparation (CDI + FMT), (ii) mice colonized with *C. albicans* then infused with human stool preparation (CDI + CA + FMT), and (iii) mice infused with human stool preparation supplemented with *C. albicans* during fecal transplantation (CDI + FMT + CA) (Fig. 5a). FMT was effective in ameliorating diarrhea, intestinal inflammation, and decreasing *C. difficile* burden, compared to CDI group, while no difference in *C. difficile* burden was observed among all groups before FMT (Fig. 5b–d). However, mice that was colonized with *C. albicans* prior to FMT or those infused with donor stool supplemented with *C. albicans* suffered significant diarrhea, intestinal inflammation, and augmented *C. difficile* burden post-FMT, when compared with mice administered with a single infusion of human stool (Fig. 5b–d). There were high levels of *C. albicans* in these recipient mice on day 1 post-FMT, though a decrease in *C. albicans* load was observed after FMT in mice colonized with *C. albicans* prior to FMT (Fig. 5e). We subsequently used an antifungal agent, fluconazole, to eradicate *C. albicans* in a group of recipient mice prior to human stool infusion (FMT) (Supplementary Figure 12a). We then compared *C. difficile* load after human stool infusion between mice with and without antifungal treatment. Antifungal treatment in recipient mice colonized with *C. albicans* before human stool infusion restored the efficacy of FMT in clearing *C. difficile* infection (Supplementary Figure 12b). These data support that *C. albicans*, either in recipient or in donor, can reduce the efficacy of FMT in clearing *C. difficile*, while antifungal treatment reestablishes its efficacy. Further research is needed to test whether this causal relationship, shown here in an animal model, applies also to patients with other diseases when doing FMT.

We also investigated the effects of *Penicillium brocae* and *Aspergillus penicillioides*, two filamentous fungi which were more enriched in healthy individuals than in CDI, on treating CDI during FMT (Supplementary Figure 13a). Mice that were colonized with *P. brocae* or *A. penicillioides* prior to FMT and mice that were supplemented with *A. penicillioides* during FMT showed higher levels of *C. difficile* burden compared with mice treated with FMT alone (Supplementary Figure 13b, on day 1 after FMT). However, these fungi-administered mice did not show any difference in symptoms of diarrhea on day 1 after FMT when compared with mice treated with FMT alone (Supplementary Figure 13c). These data suggest that different gut fungi may have disparate roles in FMT.

## Discussion

This exploratory study, to our knowledge, is the first to characterize the gut mycobiota in CDI and to elucidate mycobiota alterations after FMT in relation to treatment outcome. Patients with CDI showed enteric fungal dysbiosis. Importantly, disease recurrence after FMT was associated with persistent fungal dysbiosis, low levels of donor-derived fungal colonization, high abundance of *C. albicans* in recipient stool, and presence of *C. albicans* in donor stool. Our observation that disease cure correlates with both fungal and bacterial colonization from the donor supports the idea that the gut mycobiota may play an important role in FMT outcome. In particular, we have shown that the abundance of fecal *C. albicans*, both in recipient before treatment and in donor, correlates with FMT outcome.

The role of fungal commensals in educating the human immune system has gained new appreciation in intestinal diseases. In the steady state, bacterial communities keep fungi in check in the gut. Fungi are major causes of infections among immunocompromised or hospitalized patients with serious underlying diseases and comorbidities^24^. Commensal bacteria inhibit *C. albicans* colonization through activation of HIF-1α and LL-37^25^. Antibiotic treatment selectively and effectively eradicates the bacterial community, but consequently leads to fungal outgrowth, particularly *Candida* species^26,27^. Antibiotics or immunosuppressants are effective in the short term but they likely compromise the immune system in the longer term. A compromised immune system creates a more favorable environment to expansion of *Candida* and overgrowth of *Candida* can alter the recovery of the gut bacteria microbiota after cessation of antibiotic treatment^22,28^. Host inflammation strongly promotes *C. albicans* colonization and conversely, *C. albicans* augments inflammation in a colitis murine model^29^. *C. albicans* maintains commensalism or pathogenicity in host by modulating the local Th17/Treg balance^30^. In another aspect, it has been demonstrated that introduction of *C. albicans* into the bacterial microbiome of mice during a broad community disturbance has the potential to significantly alter the subsequent reassembly of the bacterial community as it recovers from that disturbance^28^. Taken together, our data from patients and from mouse studies support the hypothesis that an over-abundance of *Candida* species in recipients may contribute to FMT failure in CDI. Previous studies have shown that *C. albicans* and *Candida* are significantly enriched in a dextran sulfate sodium (DSS)-induced colitis mouse model as well as patients with IBD^31–33^. Antifungal treatment decreased *Candida* prevalence and ameliorated inflammatory responses in DSS colitis mice^33^. However, disruption of fungal communities by long-term use of antifungals aggravated severity of DSS colitis and allergic airway^34^. Collectively, these data support the importance of the gut fungal–bacterial homeostasis in host health, which is also consistent with our data showing that FMT nonresponders display simultaneous alterations in fungal and bacterial α-diversity when compared with responders.

In conclusion, gut mycobiota alterations in CDI patients correlate with FMT outcome, and our mouse assays support the idea that a causal relationship may exist. In particular, it is possible that *C. albicans* may contribute to CDI recurrence. Further research is needed to explore whether detailed characterization of both donor and recipient fecal mycobiomes may be useful for selection of “optimal” donors, and whether pre-FMT eradication of *C. albicans* in some recipients might increase FMT success rates in some cases.

## Methods

### Study subjects and treatment outcome

The current study was a substudy from a randomized controlled trial (RCT) of FMT versus vancomycin (STD) for patients with CDI. Consecutive CDI subjects enrolled into this RCT were invited to participate in a substudy of assessment of fecal microbiota. Patients were included if they had three or more loose or watery stools per day for at least two consecutive days or eight or more soft or loose stools in 48 h and a positive-stool test for *C. difficile* based on a two-step testing algorithm in our hospital, a positive-glutamate dehydrogenase screening test followed by a positive-polymerase chain reaction (PCR) test of *C. difficile*. A total of 31 subjects with CDI and 24 healthy household controls were recruited and stool samples at baseline were obtained for analyses of fungal and bacterial microbiomes. Among them, 24 CDI subjects consented to have stool samples collected serially after treatment for microbiome analysis. Totally, 16 CDI subjects were treated with FMT and 8 were treated with vancomycin, and they were followed up at baseline and at weeks 2, 4, 10, and 16 after treatment (Supplementary Figure 2). Subjects in FMT group received 5 days of vancomycin (125 mg, four times daily) followed by donor infused stool via nasojejunal route and those who had STD received oral vancomycin 500 mg orally four times per day for 10 days. A computer-generated randomization schedule was used to assign patients to the treatment sequences. All patients kept a stool diary and were questioned about stool frequency and consistency and medication use. The study was approved by The Joint Chinese University of Hong Kong, New Territories East Cluster Clinical Research Ethics Committee (The Joint CUHK-NTEC CREC, CREC Ref. No.: 2014.183-T; Clinical Trial registry, NCT02570477). We collected stool samples from new consecutive CDI patients. From this cohort, we included 12 CDI patients with antibiotics exposure, 12 CDI patients with no antibiotics exposure at inclusion, and an additional 17 healthy controls to determine effect of antibiotics on *C. albicans*. In addition, five new CDI subjects were treated with FMT (using two new donors with undetectable fecal *C. albicans* as determined by quantitative PCR) and consented to have stool samples collected serially after treatment for *C. albicans* quantitative analysis to confirm the findings.

Treatment response was defined as an absence of diarrhea or persistent diarrhea that could be explained by other causes with a negative stool test for *C. difficile* toxin, while relapse was defined as diarrhea with a positive-stool test for *C. difficile* toxin. Treatment cure is defined as symptom resolution and a negative *C. difficile* toxin in stool until the last follow-up (last follow-up is referred to as the last stool collection time-point, as shown in Supplementary Figure 2). Out of 16, 9 subjects who had FMT (FMT1–FMT9), and 5 of the 8 patients (STD1–STD5), who had vancomycin were cured of CDI (termed responders, Supplementary Data 3) at a median follow-up of 16 weeks. CDI recipients FMT11 and FMT12 shared the same donor, and this donor was termed “Donor11”. Clinical data of the subjects and collected stool samples are shown in Supplementary Datas 3 and 4. None of the patients had received antibiotics or proton pump inhibitors after FMT.

### Study design

#### Patient inclusion criteria

1. *C. difficile infection* was defined as diarrhea (≥3 soft, loose or watery stools per day for at least 2 consecutive days or ≥8 soft or loose stools in 48 h).
2. A positive-stool test for *C. difficile* toxin (on a two-step testing algorithm, a positive-GDH screening test followed by a positive-PCR test of *C. difficile*).
3. Age ≥ 18.
4. Written informed consent obtained.

#### Patient exclusion criteria

1. The presence of human immunodeficiency virus (HIV) infection with a CD4 count of less than 240.
2. Pregnancy.
3. Gastrointestinal bleeding.
4. Acute coronary syndrome.

#### Donor screening

Donors included individuals who are spouses or partners, first-degree relatives, other relatives, friends, and individuals unknown to the patient. They were screened with a questionnaire and excluded if they had taken antibiotics within the preceding 3 months; were on major immunosuppressive agents, including chemotherapeutic agents; had known or recent exposure to HIV, hepatitis B or C; had a current communicable disease; participated in high-risk sexual behaviors; used illicit drugs; traveled within 6 months to areas with endemic diarrheal illnesses; or had history of IBD, irritable bowel syndrome or chronic diarrhea, gastrointestinal malignancy or polyposis. In addition, donor was screened for HBsurface Ag, Anti-HBc, Anti-HCV, Anti-HIV, Syphilis EIA, stool microscopy, culture and sensitivity, stool cyst, ova, parasite, norovirus, and *C. difficile* (cytotoxin and PCR assay). All subjects and collected stool samples are listed in Supplementary Data 4.

The donors for the FMT group were healthy household controls and the donor stool samples analyzed were the same samples used for FMT. All subjects provided written informed consent.

Family members provided donor stool for subjects randomized to FMT arm. Cure after FMT or vancomycin therapy was defined as symptom resolution and negative *C. difficile* toxin in stool at last follow-up by PCR assay. Relapse was defined as diarrhea with a positive-stool test for *C. difficile* toxin.

This was a randomized, but not blinded study. However, for mycobiome and bacterial microbiome analyses on stool samples, assessments were initially performed by analysts who were blinded to the clinical outcome of the studied subjects. When the profiled mycobiome and bacterial microbiome data were available for each individual subject, correlation was then made to associate microbiome profiles with treatment outcomes of subjects.

### Infusion of donor stool

In subjects who received FMT, a nasoduodenal tube was inserted with radiology guidance. Donor feces was diluted with 500 ml of sterile saline (0.9%), blended and the supernatant was strained with filter paper and poured in a sterile bottle. Within 6 h after collection of feces by the donor, the solution was infused through a nasoduodenal tube (2–3 min per 50 ml). The tube was removed 30 min after the infusion, and patients were monitored for 2 h. In subjects who received FMT, a minimum of 50 g of donor stool was collected on the same day of infusion and used within 6 h of collection.

### Fecal DNA extraction

Fecal DNA was isolated as described below. A 100 mg fecal sample was prewashed with 1 ml ddH_2_O and pelleted by centrifugation at 10,000×*g* for 1 min. The fecal pellet was resuspended in 800 μl TE buffer (pH 7.5), supplemented with 1.6 μl 2-mercaptoethanol and 500 U lyticase (Sigma), and incubated at 37 °C for 60 min. The sample was then centrifuged at 10,000×*g* for 2 min and fecal DNA was subsequently extracted from the pellet using ZR Fecal DNA miniPrep kit (Zymo Research, Orange, CA) according to the protocal. Briefly, fecal pellet was added to the BashingBeadLysis Tube with 750 μl Lysis solution, and then processed at maximum speed for 10 min. The lysates were centrifuged at ≥10,000×*g* for 1 min. The supernatant was transferred to a Zymo-Spin^TM^ IV Spin Filter in a collection tube and centrifuged at 7000×*g* for 1 min. About 1200 μl of fecal DNA binding buffer was added to the filtrate in the collection tube, followed by concentration and purification in a new filter tube. Finally, a total of 50 μl eluted DNA with a concentration at 20–100 ng/μl was prepared for each sample.

### Fungal ITS2 sequencing and quality control

The final fecal DNA for fungal sequencing was amplified based upon internal transcribed spacer 2 (ITS2) region using primers as below and PrimeSTAR HS DNA Polymerase kit (TaKaRa, Japan). The primer pairs are ITS2-F: 5′-GCATCGATGAAGAACGCAGC-3′, ITS2-R: 5′-TCCTCCGCTTATTGATATGC-3′. ITS2 amplicons were generated with 38 cycles of 3-step PCR: 98 °C 10 s, 59 °C 10 s, and 72 °C 30 s. PCR samples were then sequenced on the Illumina MiSeq PE300 platform (2 × 300 bp, BGI, China), 151,524 ± 97,694 (mean ± SD) clean sequences obtained on average (sequence statistics in Supplementary Data 5).

Raw reads were filtered by SOAPnuke (v 1.5.3) (http://soap.genomics.org.cn/) developed by BGI as follows: (i) adapters removed, (ii) read removed if N base is more than 3% of the read, (iii) read removed if bases with quality low than 20 were more than 40% of read, and (iv) all duplicates removed. Quality control and data analysis were further implemented in PIPITS (v 1.4.5)^35^. Briefly, PIPITS_PREP prepares raw reads from Illumina MiSeq sequencers for ITS extraction; PIPITS_FUNITS extracts ITS2 from the reads; and PIPITS_PROCESS analyses the reads to produce OTU abundance tables and the Ribosomal Database Project (RDP) taxonomic assignment table for downstream analysis. The quality trimmed and ITS2 extracted reads were aligned to fungi UNITE database exploiting RDP classifier 2.10 for taxonomic assignment to produce OTU abundance table (based on sequence identity ≥ 97% identity) and phylotype abundance tables at different taxonomic levels, for downstream analysis.

The fungal OTU and phylotype abundance data were imported into *R 3.2.3*. Richness, diversity, and evenness calculation were performed using the estimated richness function of the phyloseq package. Spearman correlation and their significance were calculated using the *cor* and *cor.test* functions in R, respectively. For the fungal–bacterial taxa comparisons, Spearman correlations were calculated for the relative abundance of the differentially presented fungal taxa and the bacterial taxa determined to be significantly associated with disease by Lefse analysis. Correlation plots were generated using the corrplot package in R. Heatmaps were generated using the *pheatmap* package in R.

### Quantitative PCR for detection of total fungal load in human fecal DNA samples

Total fungal loads in human stools were quantified by TaqMan qPCR analysis (Premix Ex Taq^TM^, TaKaRa) of extracted human fecal DNA using primers^36^: *Fungi-quant*-F 5′-GGRAAACTCACCAGGTCCAG-3′; *Fungi-quant*-R 5′-GSWCTATCCCCAKCACGA-3′, and probe: 5′-TGGTGCATGGCCGTT-3′.

### Quantitative PCR for detection of *C. albicans* in human fecal DNA samples

*C. albicans* loads in human stools were quantified by qPCR analysis (SsoAdvanced SYBR Green Supermix, Bio-Rad) of extracted human fecal DNA using *C. albicans* specific primers: *C.albicans*-F 5′-CCTGTTTGAGCGTCGTTTCTC-3′; *C. albicans*-R 5′-TTTGGTTAGACCTAAGCCATTGTCA-3′. *C. albicans* absolute abundance was determined using standard curve constructed with reference genomic DNA (gDNA) of *C. albicans*.

### LefSe linear discriminant analysis and multivariate analysis

To compare differences in the configurations of fungal and bacterial microbiomes between CDI patients and healthy controls, between FMT responders and nonresponders, between FMT treatment and vancomycin (STD) treatment, LefSe analyses were performed on the Huttenhower lab Galaxy server (http://huttenhower.sph.harvard.edu/galaxy/) by importing the fungal and bacterial relative abundance values and associated sample metadata, with FDR-adjusted *q* value < 0.05 considered significant and effect size calculated. MaAsLin (Multivariate Analysis by Linear Models) was implemented to identify associations between clinical metadata (age, gender, household, CDI severity, and underlying IBD) and fungal community abundance matrix on the Huttenhower lab Galaxy server (http://huttenhower.sph.harvard.edu/galaxy/).

### Nonmetric multidimensional scaling analysis

The difference in fecal fungal community structures between controls and CDI was performed via NMDS plot based upon Bray–Curtis dissimilarities among all subjects. The two ellipses correspond to the fecal mycobiota community dispersion range within each group, controls and CDI, respectively, drawn using the function *veganCovEllipse* in the Package *Vegan* in *R* basing upon the Least-Squares criterion for estimation of the best *fit* to an *ellipse* from a given set of mycobiota communities.

### Bacterial 16S rRNA sequencing and data analysis

The final fecal DNA samples were subject to bacterial 16S rRNA V4 region amplification and sequenced on the Illumina MiSeq PE250 platform (2 × 250 bp, BGI, China), 132,081 ± 65,429 (number ± SD) sequences obtained on average (sequence statistics in Supplementary Data 6). Quality control and data analysis were implemented in mothur (v 1.38.0) as previously described^37^. Any sequences with ambiguous bases and anything longer than 275 bp were removed, and aligned against the nonredundant Greengenes database (v 13.8)^38^ using the NAST algorithm. Any sequences that failed to align with the V3–4 region were discarded. The remaining sequences were trimmed to the same alignment coordinates over which they fully overlapped, followed by removal of homopolymers and detection for the presence of chimeras by UChime.

The resulting sequences were classified against the Greengenes database and annotated with deepest level taxa represented by pseudo-bootstrap confidence scores of at least 80% averaged over 1000 iterations of the naive Bayesian classifier. Any sequences that were classified as either being originated from archaea, eukarya, chloroplasts, mitochondria, or unknown kingdoms, were removed. The annotated sequences were assigned to phylotypes according to their consensus taxonomy with which at least 80% of the sequences agreed. Closed reference OTUs sharing 97% identity were clustered as well and assigned taxonomy according to the Greengenes database. LefSe analysis was performed to define bacterial taxa associated with CDI and healthy controls. The relative abundance of these abundance-differential taxa identified by LefSe in pre-FMT baseline samples and post-FMT last follow-up samples were plotted using *pheatmap* R package.

### Calculation of donor transferred OTUs in recipients

In samples after FMT, if a fungal or bacterial OTU was not present in the recipient baseline sample but present both in the corresponding donor baseline sample and in the recipient post-FMT sample, the OTU was defined as “donor derived”; if an OTU was not present in the corresponding donor baseline sample, but detected both in the recipient baseline sample and in the recipient post-FMT sample, the OTU was defined as “recipient exclusive”; if an OTU was present across the recipient baseline sample, the recipient post-FMT sample and the corresponding donor baseline sample, the OTU was defined as “donor–recipient co-existed”.

### Mouse husbandry and model of *C. difficile* infection

Studies were conducted on 4- to 6-week-old female C57BL/6 that were reared in groups of nine. Individual mice were randomized after arrival. Mice were subjected to a previously described model of CDI^39^. Briefly, mice were given an antibiotic cocktail of kanamycin (0.4 mg/mL), gentamicin (0.035 mg/mL), colistin (850 U/mL), metronidazole (0.215 mg/mL), and vancomycin (0.045 mg/mL) (all antibiotics were purchased from Sigma–Aldrich, St. Louis, MO) in their drinking water for 3 days. Mice were then given 2 days of recovery before administration of 10^7^ spores of *C. difficile* (strain HK31423, Ribotype 002, a clinical isolate during an outbreak at Prince of Wales Hospital in Hong Kong) in phosphate-buffered saline (PBS) via oral gavage. Animal grouping and research scheme were designed as shown in Fig. 4a. On day 1 poststool infusion, diarrhea was evaluated by stool water content, calculated as stool weight loss after air drying at 70 °C for 4 h. Cecums were harvested, fixed in 4% formalin solution and embedded in paraffin on day 1 after FMT. Sections were stained with hemotoxylin and eosin for histological assessment^40^. In experiment for investigating the effect of vancomycin administration on the presence of *Candida* in mice, seven 4- to 6-week old female C57BL/6 mice were given vancomycin (0.1 mg/mL) in their drinking water for 3 days. Stool samples before and after vancomycin regime were collected and subsequently determined for *Candida* levels by qPCR analysis (SsoAdvanced SYBR Green Supermix, Bio-Rad) using primers^41^: *pan-Candida*-F 5′-GCAAGTCATCAGCTTGCGTT-3′; *pan-Candida*-R 5′-TGCGTTCTTCATCGATGCGA-3′. All animal experiments were approved and performed in compliance with the Animal Experimentation Ethics Committee (AEEC) of The Chinese University of Hong Kong.

### *C. albicans* administration and donor stool infusion in mice

*C. albicans* (10231, from ATCC, USA) was administered to mice (2 × 10^8^ cfu per mouse) via gavage after 3-day antibiotic treatment or supplemented in donor stool slurry at the time of donor stool infusion. Human stool from a healthy volunteer (Chinese, male, age 28 years), without presence of *C. albicans* as measured by qPCR, was obtained with informed consent. For stool microbiota infusions, approximately 2 g of stool samples were cut in an anaerobic chamber and suspended in 10 ml of PBS. Mice were colonized by oral gavage of 150 μl of fecal slurry with or without supplementation of *C. albicans* on day 2 after *C. difficile* challenge. For antifungal experiment, animal grouping and research scheme were designed as shown in Supplementary Figure 12a. Mice was initially colonized with *C. albicans* (2 × 10^8^ cfu per mouse) after 3 days of antibiotic cocktail treatment in the drinking water, followed by 4 days of fluconazole treatment supplemented in the drinking water (0.5 mg/mL, Sigma). Then the mice were subjected to *C. difficile* administration (10^7^ spores per mice) through gavage after a consecutive 1.5-day antibiotic cocktail- and 1.5-day free water-drinking. Human stool infusion was performed 2 days later after *C. difficile* gavage. Both *C. difficile* load and *C. albicans* load were enumerated by cultivation on day 0 before FMT and day 1 after FMT.

### Quantification of *C. difficile* and *C. albicans* burdens in mouse feces

Mouse stool were collected both before and after stool infusion. Fecal *C. difficile and C. albicans burdens* on day 0 before and day 1 after stool infusion were measured by cultivation. Samples were diluted in PBS and, respectively, plated on taurocholate cycloserine cefoxitin fructose agar for quantification of *C. difficile* burden, on Sabouraud dextrose agar (SDA) for quantification of *C. albicans* load. Stool samples prior to *C. albicans* colonization from antibiotic-treated mice were plated on SDA to ensure that mice were *C. albicans* culture negative.

### Administration of *P. brocae*, and *A. penicillioides*, and donor stool infusion in mice

A 4- to 6-week-old female C57BL/6 that were reared in groups of eight. Animal grouping and research scheme were designed as shown in Supplementary Figure 13a. *P. brocae* (CBS116042, from Westerdijk Fungal Biodiversity Institute, Netherlands) and *A. penicillioides* (CBS112373, from Westerdijk Fungal Biodiversity Institute, Netherlands) were administered to mice, respectively (approximately 0.5 g/kg), via gavage after 3-day antibiotic treatment or supplemented in donor stool slurry at the time of donor stool infusion. Colonization of *P. brocae* or *A. penicillioides* in mice was confirmed by PCR with detectable genomic DNA of respective species in fecal DNA after oral administration of each fungus. For stool microbiota infusions, approximately 2 g of stool samples were cut in an anaerobic chamber and suspended in 10 ml of PBS. Mice were colonized by oral gavage of 150 μl of fecal slurry with or without supplementation of these fungi on day 2 after *C. difficile* challenge.

## Electronic supplementary material

Supplementary Information

Description of Additional Supplementary Files

Supplementary Data 1

Supplementary Data 2

Supplementary Data 3

Supplementary Data 4

Supplementary Data 5

Supplementary Data 6

## Data Availability

Sequence data and accompanying metadata have been deposited to the NCBI Sequence Read Archive under BioProject accession numbers PRJNA419097 and PRJNA419104.

## References

[1] Smits LP, Bouter KEC, de Vos WM, Borody TJ, Nieuwdorp M (2013). Therapeutic potential of fecal microbiota transplantation. Gastroenterology.

[2] Vrieze A (2012). Transfer of intestinal microbiota from lean donors increases insulin sensitivity in individuals with metabolic syndrome. Gastroenterology.

[3] van Nood E (2013). Duodenal infusion of donor feces for recurrent *Clostridium difficile*. New Engl. J. Med..

[4] Lee CH (2016). Frozen vs. fresh fecal microbiota transplantation and clinical resolution of diarrhea in patients with recurrent *Clostridium difficile* infection: a Randomized Clinical Trial. J. Am. Med. Assoc..

[5] Drekonja D (2015). Fecal microbiota transplantation for *Clostridium difficile* infection: a systematic review. Ann. Intern. Med..

[6] Paramsothy S (2017). Faecal microbiota transplantation for inflammatory bowel disease: a systematic review and meta-analysis. J. Crohns Colitis.

[7] Costello SP (2017). Systematic review with meta-analysis: faecal microbiota transplantation for the induction of remission for active ulcerative colitis. Aliment. Pharmacol. Ther..

[8] Kelly CR, Kahn SA, Kashyap P (2015). Update on fecal microbiota transplantation 2015: indications, methodologies, mechanisms, and outlook (vol 149, p. 223, 2015). Gastroenterology.

[9] Khoruts A, Sadowsky MJ (2011). Therapeutic transplantation of the distal gut microbiota. Mucosal Immunol..

[10] Manichanh C (2010). Reshaping the gut microbiome with bacterial transplantation and antibiotic intake. Genome Res..

[11] Li SS (2016). Durable coexistence of donor and recipient strains after fecal microbiota transplantation. Science.

[12] Khoruts A, Sadowsky MJ (2016). Understanding the mechanisms of faecal microbiota transplantation. Nat. Rev. Gastroenterol. Hepatol..

[13] Zuo T (2017). Bacteriophage transfer during faecal microbiota transplantation in *Clostridium difficile* infection is associated with treatment outcome. Gut.

[14] Chehoud C (2016). Transfer of viral communities between human individuals during fecal microbiota transplantation. mBio.

[15] Broecker F (2016). Long-term changes of bacterial and viral compositions in the intestine of a recovered *Clostridium difficile* patient after fecal microbiota transplantation. Mol. Case Stud..

[16] Limon JJ, Skalski JH, Underhill DM (2017). Commensal fungi in health and disease. Cell Host Microbe.

[17] Iliev ID, Leonardi I (2017). Fungal dysbiosis: immunity and interactions at mucosal barriers. Nat. Rev. Immunol..

[18] Jiang TT (2017). Commensal fungi recapitulate the protective benefits of intestinal bacteria. Cell Host Microbe.

[19] Leffler DA, Lamont JT (2015). *Clostridium difficile* infection. New Engl. J. Med.

[20] Stevens V, Dumyati G, Fine LS, Fisher SG, Van Wijngaarden E (2011). Cumulative antibiotic exposures over time and the risk of *Clostridium difficile* infection. Clin. Infect. Dis..

[21] Kobayashi-Sakamoto M, Tamai R, Isogai E, Kiyoura Y (2018). Gastrointestinal colonisation and systemic spread of *Candida albicans* in mice treated with antibiotics and prednisolone. Microb. Pathog..

[22] Mason KL (2012). *Candida albicans* and bacterial microbiota interactions in the cecum during recolonization following broad-spectrum antibiotic therapy. Infect. Immun..

[23] Leffler DA, Lamont JT (2015). *Clostridium difficile* infection. New Engl. J. Med..

[24] Flevari A, Theodorakopoulou M, Velegraki A, Armaganidis A, Dimopoulos G (2013). Treatment of invasive candidiasis in the elderly: a review. Clin. Interv. Aging.

[25] Fan D (2015). Activation of HIF-1 alpha and LL-37 by commensal bacteria inhibits *Candida albicans* colonization. Nat. Med..

[26] Mason KL (2012). Interplay between the gastric bacterial microbiota and *Candida albicans* during postantibiotic recolonization and gastritis. Infect. Immun..

[27] Dollive S (2013). Fungi of the murine gut: episodic variation and proliferation during antibiotic treatment. PLoS One.

[28] Downward JRE, Falkowski NR, Mason KL, Muraglia R, Huffnagle GB (2013). Modulation of postantibiotic bacterial community reassembly and host response by *Candida albicans*. Sci. Rep..

[29] Jawhara S (2008). Colonization of mice by *Candida albicans* is promoted by chemically induced colitis and augments inflammatory responses through galectin-3. J. Infect. Dis..

[30] Bonifazi P (2009). Balancing inflammation and tolerance in vivo through dendritic cells by the commensal *Candida albicans*. Mucosal Immunol..

[31] Sokol H (2017). Fungal microbiota dysbiosis in IBD. Gut.

[32] Sartor RB, Wu GD (2017). Roles for intestinal bacteria, viruses, and fungi in pathogenesis of inflammatory bowel diseases and therapeutic approaches. Gastroenterology.

[33] Iliev ID (2012). Interactions between commensal fungi and the C-type lectin receptor dectin-1 influence colitis. Science.

[34] Wheeler ML (2016). Immunological consequences of intestinal fungal dysbiosis. Cell Host Microbe.

[35] Gweon HS (2015). PIPITS: an automated pipeline for analyses of fungal internal transcribed spacer sequences from the Illumina sequencing platform. Methods Ecol. Evol..

[36] Liu CM (2012). FungiQuant: a broad-coverage fungal quantitative real-time PCR assay. BMC Microbiol..

[37] Schloss PD (2009). Introducing mothur: open-source, platform-independent, community-supported software for describing and comparing microbial communities. Appl. Environ. Microb..

[38] McDonald D (2012). An improved Greengenes taxonomy with explicit ranks for ecological and evolutionary analyses of bacteria and archaea. ISME J..

[39] Chen XH (2008). A mouse model of *Clostridium difficile*-associated disease. Gastroenterology.

[40] Erben U (2014). A guide to histomorphological evaluation of intestinal inflammation in mouse models. Int. J. Clin. Exp. Pathol..

[41] Zhang J (2016). Development of Candida-specific real-time PCR assays for the detection and identification of eight medically important Candida species. Microbiol. Insights.

