# Supplementary material for: Gut fungal dysbiosis correlates with reduced efficacy of fecal microbiota transplantation in Clostridium difficile infection
Source: Nat Commun. 2018 Sep 10;9:3663. doi: 10.1038/s41467-018-06103-6 (PMC6131390; doi:10.1038/s41467-018-06103-6)
Supplement: Supplementary file 1 — Supplementary Information [file 41467_2018_6103_MOESM1_ESM.pdf]

Supplementary Information

Gut fungal dysbiosis correlates with reduced efficacy of fecal microbiota transplantation in *Clostridium difficile* infection

Zuo, Ng et al.

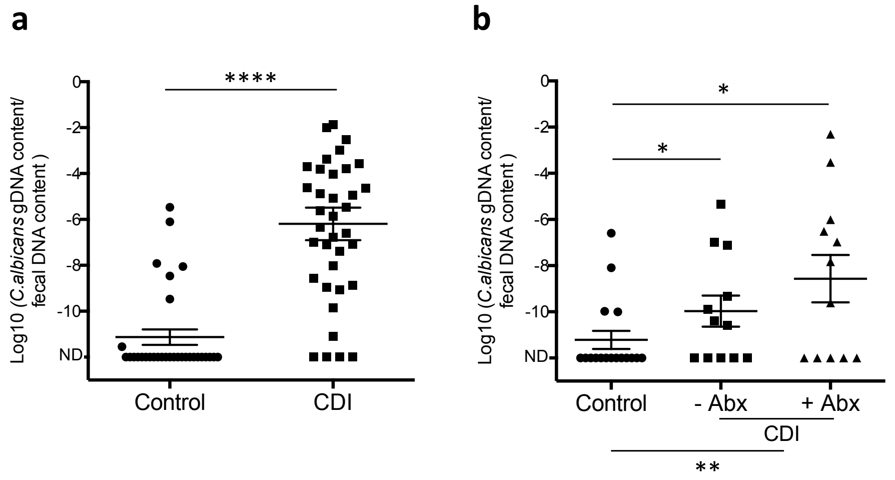

Supplementary Figure 1. Quantification of fecal *C. albicans* levels in CDI subjects and healthy controls by qPCR. a, qPCR detection of *C. albicans* on CDI subjects and healthy controls from the discovery cohort. Comparison of the fecal *C. albicans* level between control and CDI was determined by Mann-Whitney test, ****P < 0.0001. **b**, qPCR detection of *C. albicans* on an additional set of subjects (17 healthy individuals, 12 CDI subjects with and 12 without antibiotic use at inclusion). Statistical significance was tested by unpaired Mann-Whitney test. * P<0.05, ** P<0.01. Graphs are shown in mean±s.e.m. ND denotes no detectable *C. albicans* in the feces as determined by quantitative PCR.

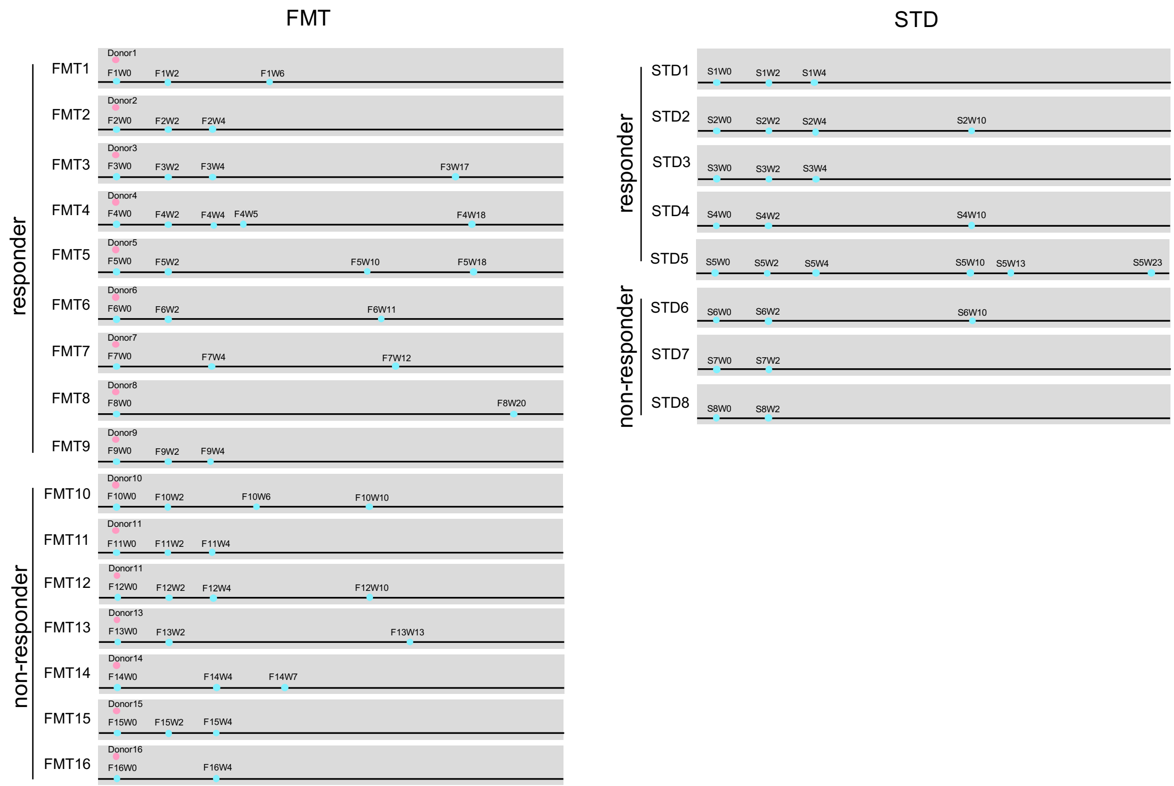

Supplementary Figure 2. Longitudinal timeline of stool sample collection (expressed in weeks). “F” indicates FMT treated subject. “Donor” indicates FMT donor. “S” indicates subject treated with standard therapy (STD, vancomycin). “W” indicates weeks post treatment. Red dots indicate donor samples, green dots indicate FMT recipient samples sampled at different time points.

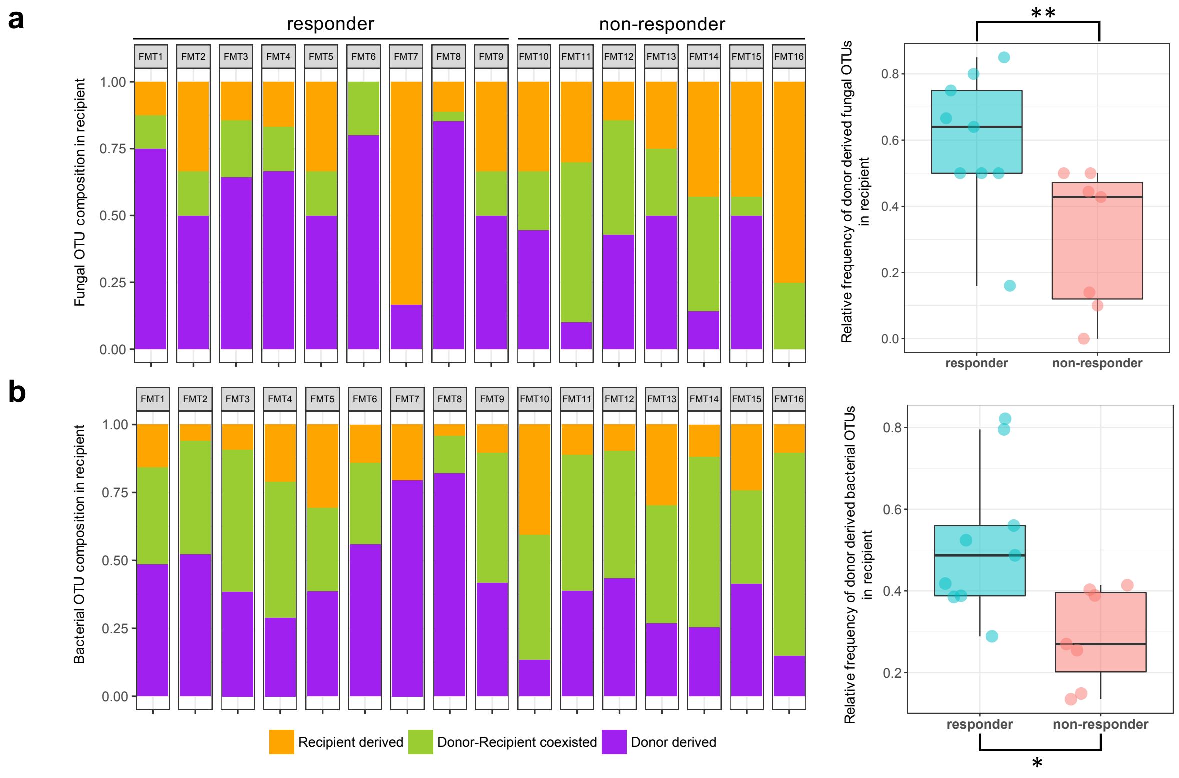

Supplementary Figure 3. Colonization of donor-derived fungal and bacterial taxa in FMT recipients in association with treatment response. a, Presence of fungal OTUs in FMT recipients at the last follow-up. The color of the bar indicates the origin of the fungal OTUs in the recipient. Purple indicates donor-derived OTUs colonized in the recipient, orange indicates OTUs exclusively present in recipient at baseline but not in donor at baseline, while green indicates OTUs present both in donor and in recipient before FMT. Comparison of the relative frequency of donor derived bacterial OTUs in FMT responders and in non-responders is shown. Statistical significance was determined by Mann-Whitney test, $^{**}P < 0.01$. **b**, Presence of bacterial OTUs in FMT recipients at the last follow-up. Comparison of the relative frequency of donor-derived bacterial OTUs in FMT responders and in non-responders is shown. Statistical significance was determined by Mann-Whitney test, $^{*}P < 0.05$.

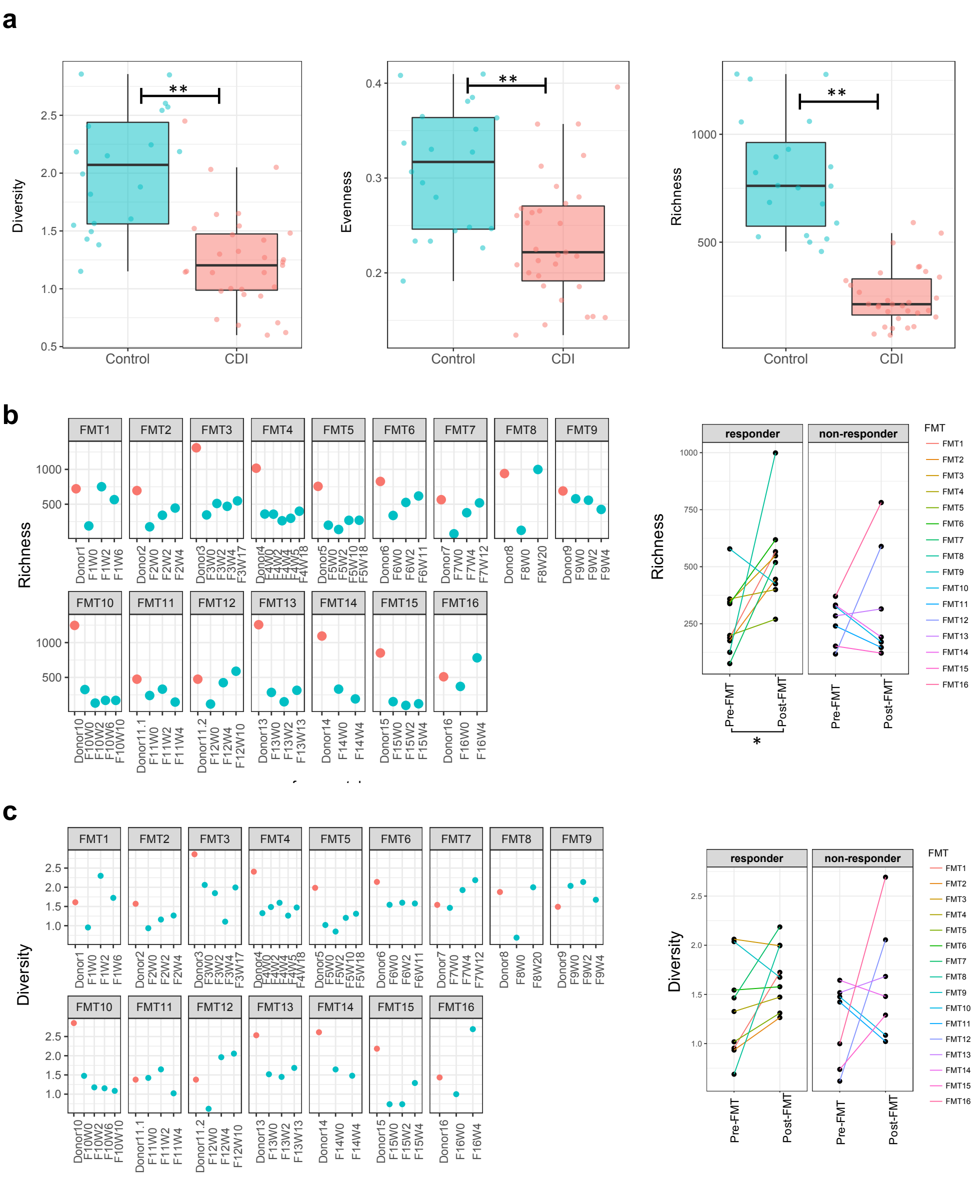

Supplementary Figure 4. Post-FMT alterations in the fecal bacterial diversity and richness of CDI recipients in association with FMT response. a, Comparison of the fecal bacterial Shannon diversity, evenness, chao1 richness in healthy controls and in CDI subjects. The boxes extend from the 1st to 3rd quartile, with the median depicted by a vertical line. The dots indicate individual values of the studied subjects. Statistical significance was determined by Mann-Whitney test, $^{**}P < 0.01$. Fecal bacterial richness (**b**) and diversity (**c**) alterations in FMT recipients over the course of longitudinal follow-up and in their corresponding donors at baseline. Comparison of the fungal richness and diversity of pre-FMT samples and post-FMT samples collected at the last follow-up are shown in FMT responders and FMT non-responders respectively. Statistical significance was determined by paired Wilcoxon signed rank test, $^{*}P < 0.05$. “F” indicates FMT treated subject. “W” indicates weeks post treatment.

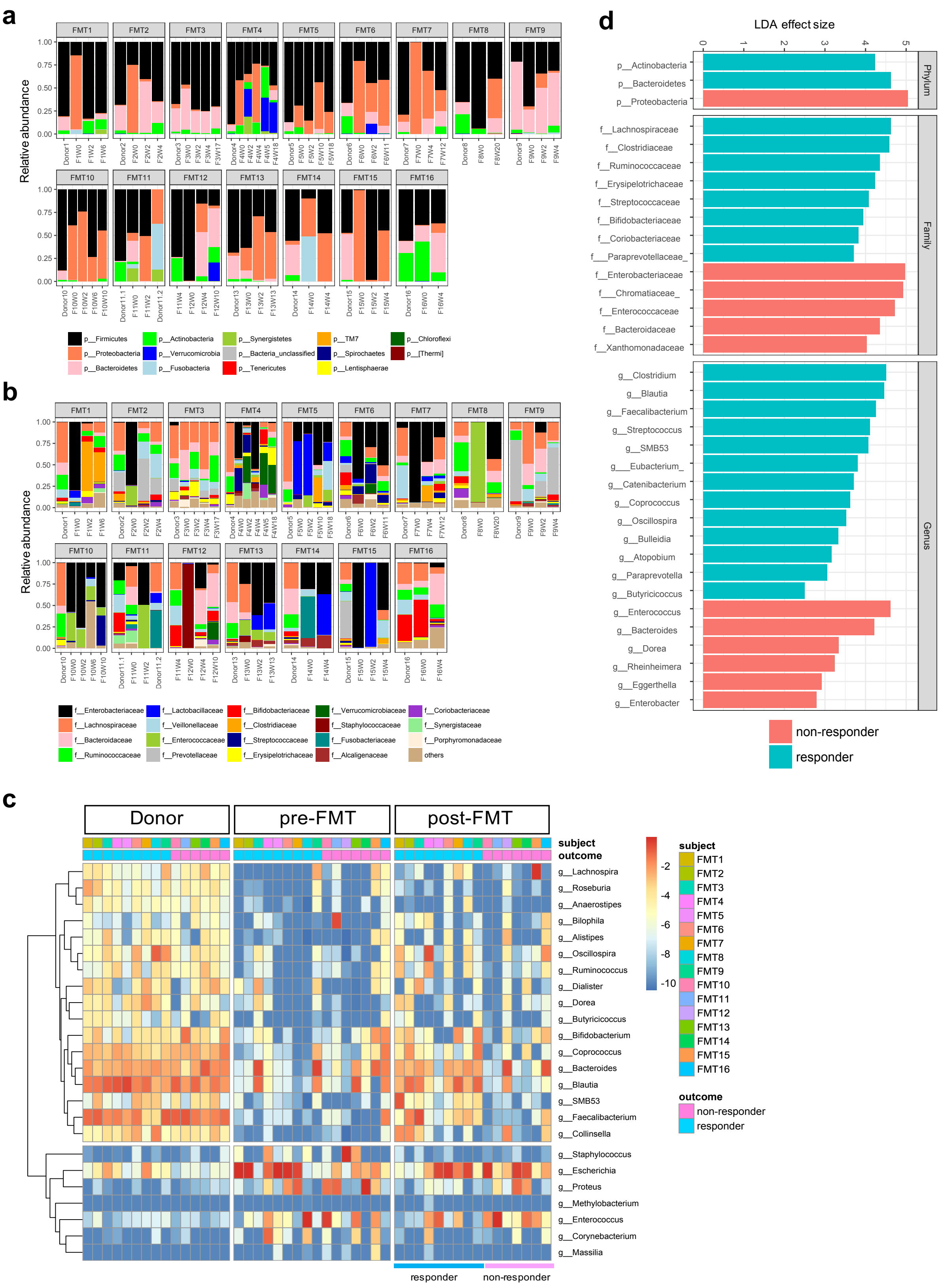

Supplementary Figure 5. Post-FMT alterations in the taxonomic composition of the bacterial microbiota of CDI recipients in association with FMT response. Bacterial configurations in FMT recipients over the course of longitudinal follow-up and

in their corresponding donors at baseline, at the phylum (**a**) and family (**b**) levels. **c**, Heatmap of the relative abundance of differentially presented bacterial genera in donor, pre-FMT and post-FMT last follow-up samples. **d**, Differentially presented bacteria taxa across post-FMT samples of FMT responders versus non-responders at the phylum, family, and genus levels. Statistical significance level was determined by LefSe analysis with FDR correction (only those taxa with q values < 0.05 and LDA effect size >2 are shown).

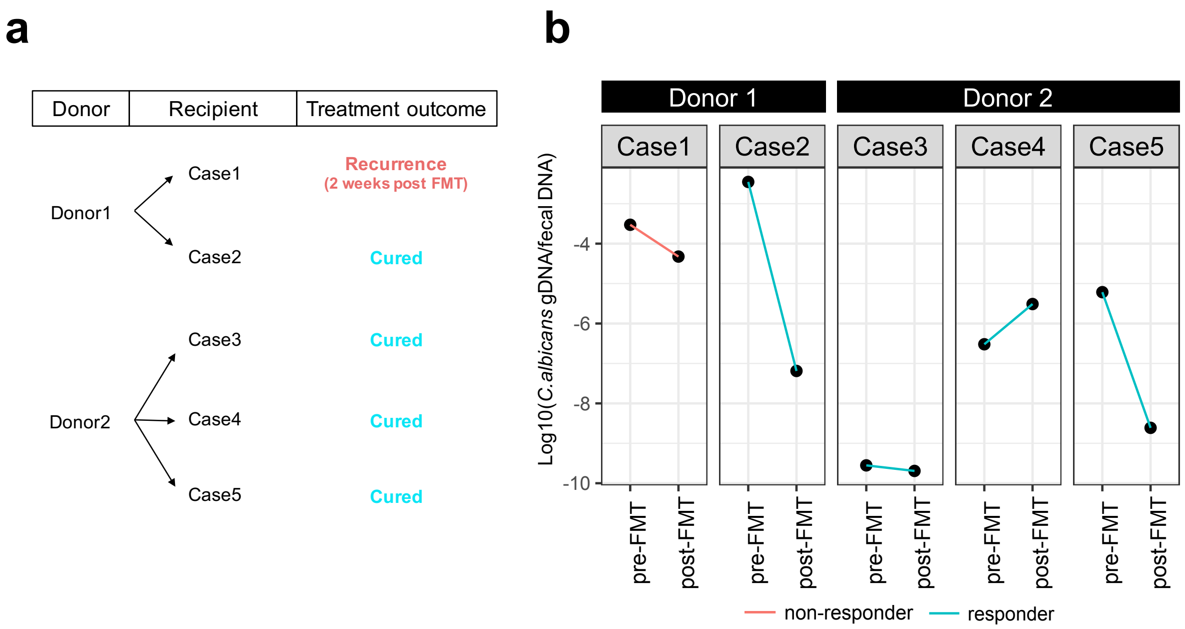

Supplementary Figure 6. Quantification of fecal *C. albicans* levels on FMT subjects in the initial cohort and 5 subsequent new CDI cases after FMT. a, Schematic design of 5 new FMT cases and their treatment outcomes; 2 donors with undetectable fecal *C. albicans* as determined by qPCR were screened and selected for donor stool infusion in these new FMT cases. **b**, Alterations in the fecal *C. albicans* in recipients after FMT in these 5 new FMT recipients. Fecal *C. albicans* levels were determined by qPCR.

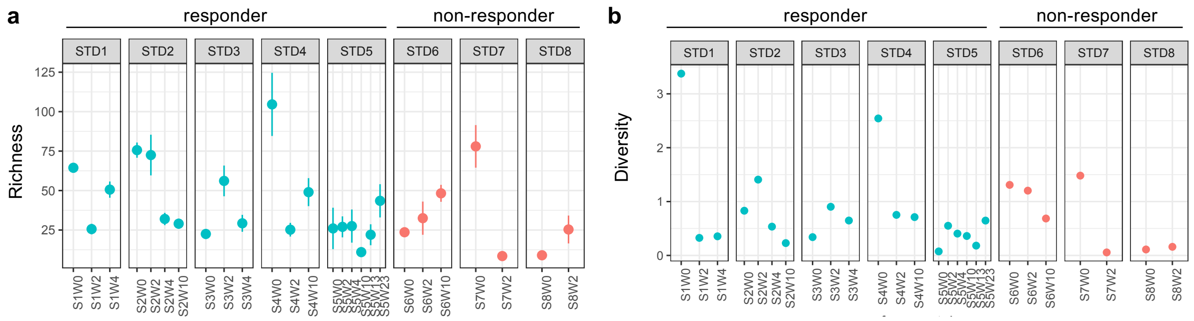

c

Post-treatment alteration in fungal diversity

	FMT	STD
Individuals increased in diversity	13	3
Individuals decreased in diversity	3	5

Chi-square test: *p*=0.032 *

Post-treatment alteration in fungal richness

	FMT	STD
Individuals increased in richness	11	3
Individuals decreased in richness	5	5

Chi-square test: *p*=0.371

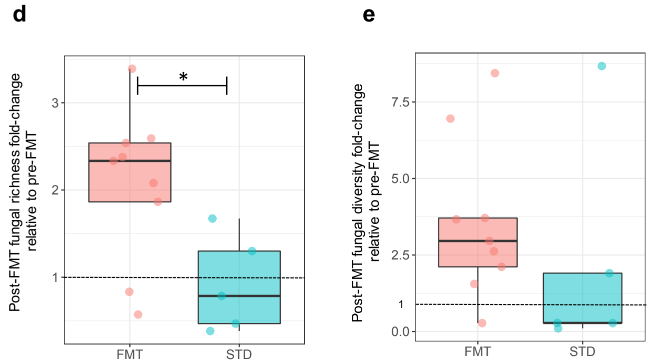

Supplementary Figure 7. Post-antibiotic alterations in the fecal mycobiota of CDI subjects treated with vancomycin in association with treatment response. Fecal fungal richness (**a**) and diversity (**b**) alterations over the course of longitudinal follow-up in CDI subjects who received vancomycin treatment. “S” indicates CDI subject received vancomycin treatment (standard therapy, STD). “W” indicates weeks post vancomycin treatment. **c**, Frequencies of CDI individuals with increased or decreased fungal diversity and richness post treatment with respect to FMT and STD treatment. Statistical significance was determined by *Chi*-square test, *$P < 0.05$. Comparison of post-FMT fold change (FC) in the fecal mycobiota richness (**d**) and diversity (**e**) relative to their corresponding pre-FMT levels in FMT responders and STD responders. Statistical significance was determined by t test, *$P < 0.05$.

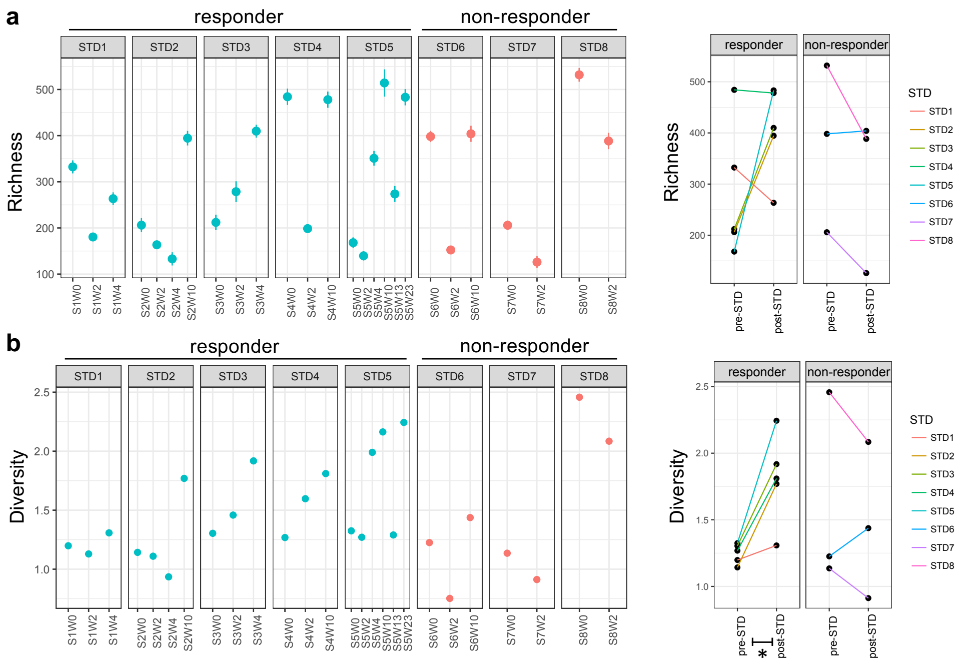

Supplementary Figure 8. Fecal bacterial microbiota richness (a) and diversity (b) alterations in STD subjects over the course of longitudinal follow-up. "S" indicates vancomycin treated subject (STD treatment). "W" indicates weeks post treatment. Comparisons between pre-STD and post-STD samples were performed by paired Wilcoxon signed rank test, *$P < 0.05$.

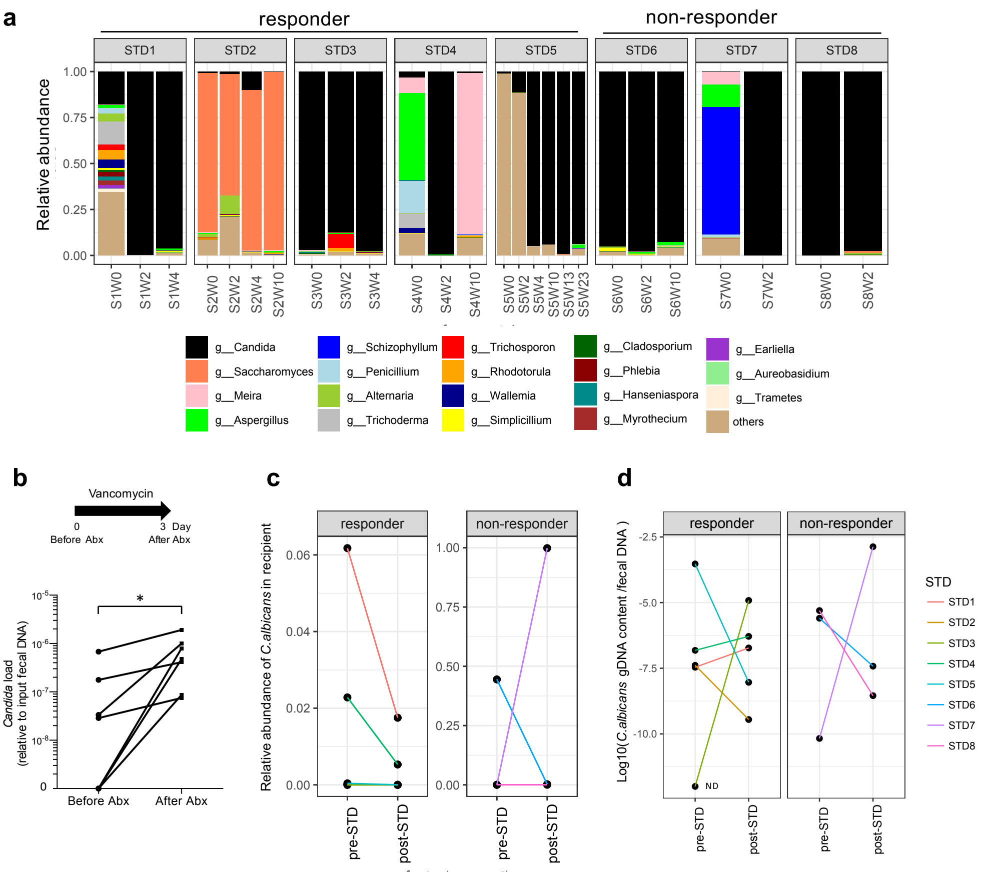

Supplementary Figure 9. Post-STD alterations in the fecal fungal composition of CDI subjects on vancomycin treatment. a, Alterations in the fecal fungal composition at the genus level in CDI subjects on vancomycin regime at different time points up to the last follow-up. **b**, Alterations of the fecal Candida level in mice after 3-day vancomycin treatment. n=7. Statistical significance was determined by paired Wilcoxon signed rank test, **P* < 0.05. Alterations of the relative abundance (**c**) and absolute abundance (**d**) of fecal *C. albicans* after vancomycin treatment in CDI subjects. ND denotes no detectable *C. albicans* in the feces.

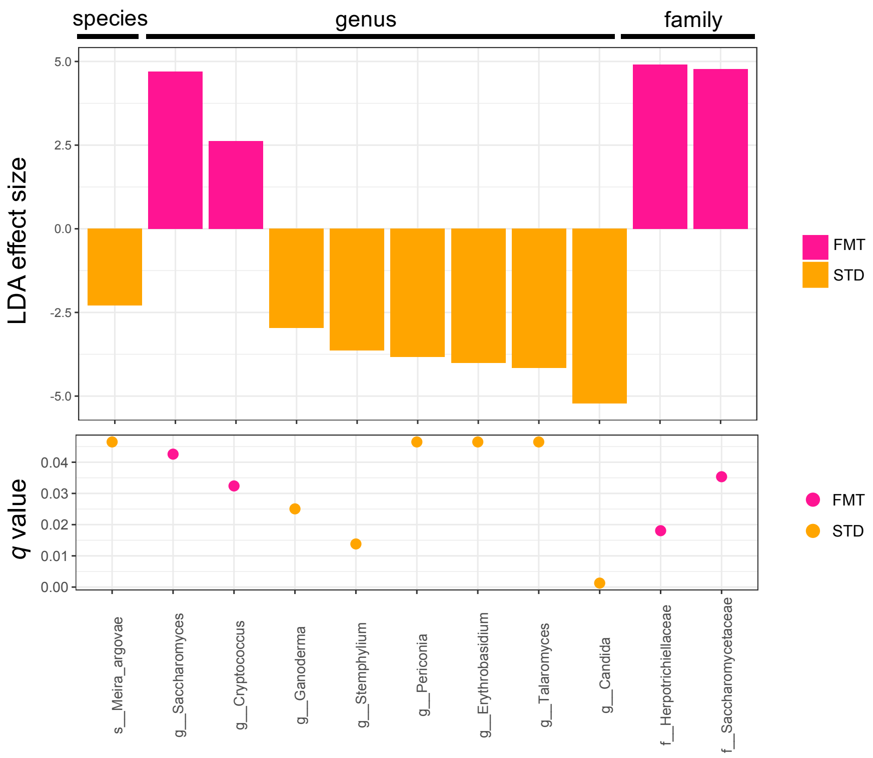

Supplementary Figure 10. Differentially enriched fungal taxa across all post-treatment samples of FMT subjects versus STD subjects at the family, genus and species levels. Statistical significance level was determined by LefSe analysis with FDR correction (only those taxa with q values < 0.05 and LDA effect size >2 are shown).

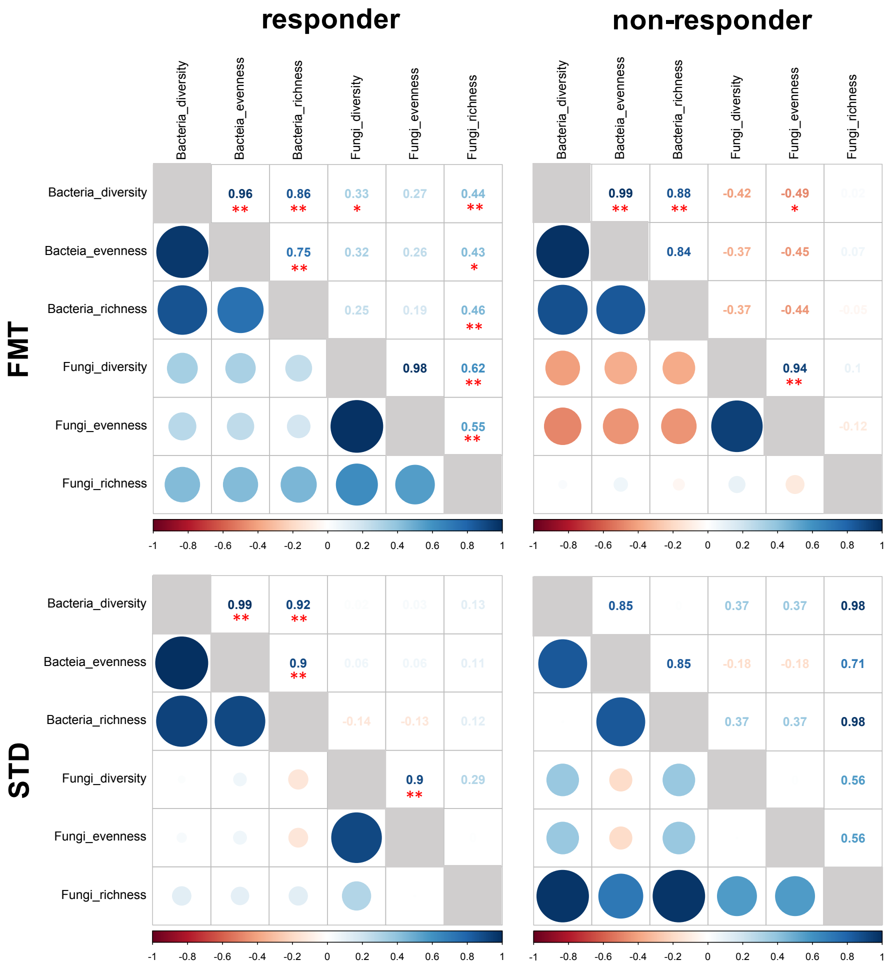

Supplementary Figure 11. Spearman correlations between fungal diversity, evenness, richness and bacterial diversity, evenness, richness, with respect to FMT and STD treatment and treatment response. Statistical significance was determined for all pairwise comparisons; significant correlations (*P* value < 0.05) are displayed with asterisk. Blue circles and positive values indicate positive correlations, red circles and negative values indicate inverse correlations. The size and shading indicate the magnitude of the correlation where darker shades are more intensively correlated than lighter ones.

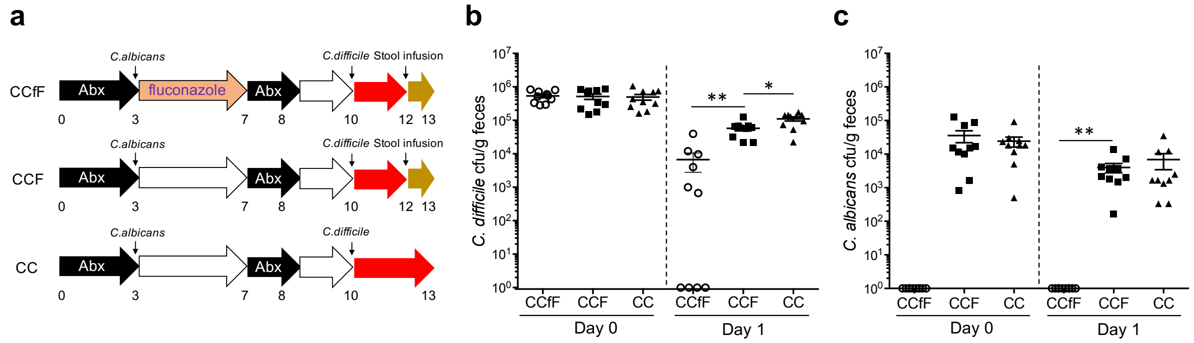

Supplementary Figure 12. Pre-FMT eradication of *C. albicans* in recipient mice restores FMT efficacy in clearing *C. difficile* infection. a, Schematic diagram of antifungal treatment and stool infusion (FMT) in a murine model with di-colonisation of *C. albicans* and *C. difficile*. Antifungal (fluconazole) treatment was ceased at day 4 after administration of *C. albicans* when *C. albicans* was determined negative by cultivation. "CCfF", mouse group with di-colonisation of *C. albicans* and *C. difficile* and treatments of fluconazole and FMT; "CCF", mouse group with di-colonisation of *C. albicans* and *C. difficile* and treatment of FMT; "CC", mouse group with di-colonisation of *C. albicans* and *C. difficile*. **b**, Enumeration of *C. difficile* in feces of mice on day 0 before FMT and day 1 post FMT (n=10 mice per group). Statistical significance was determined by unpaired Mann-Whitney test. * *P* <0.05, ** *P* <0.01. **c**, Enumeration of *C. albicans* in feces of mice both on day 0 before FMT and day 1 post FMT (n=10 mice per group). Statistical significance represents comparison between *C. albicans* load on day 0 before FMT and day 1 post FMT, by paired Mann-Whitney test. ** *P* <0.01. Dot graphs show mean$\pm$s.e.m, performed at least two times independently.

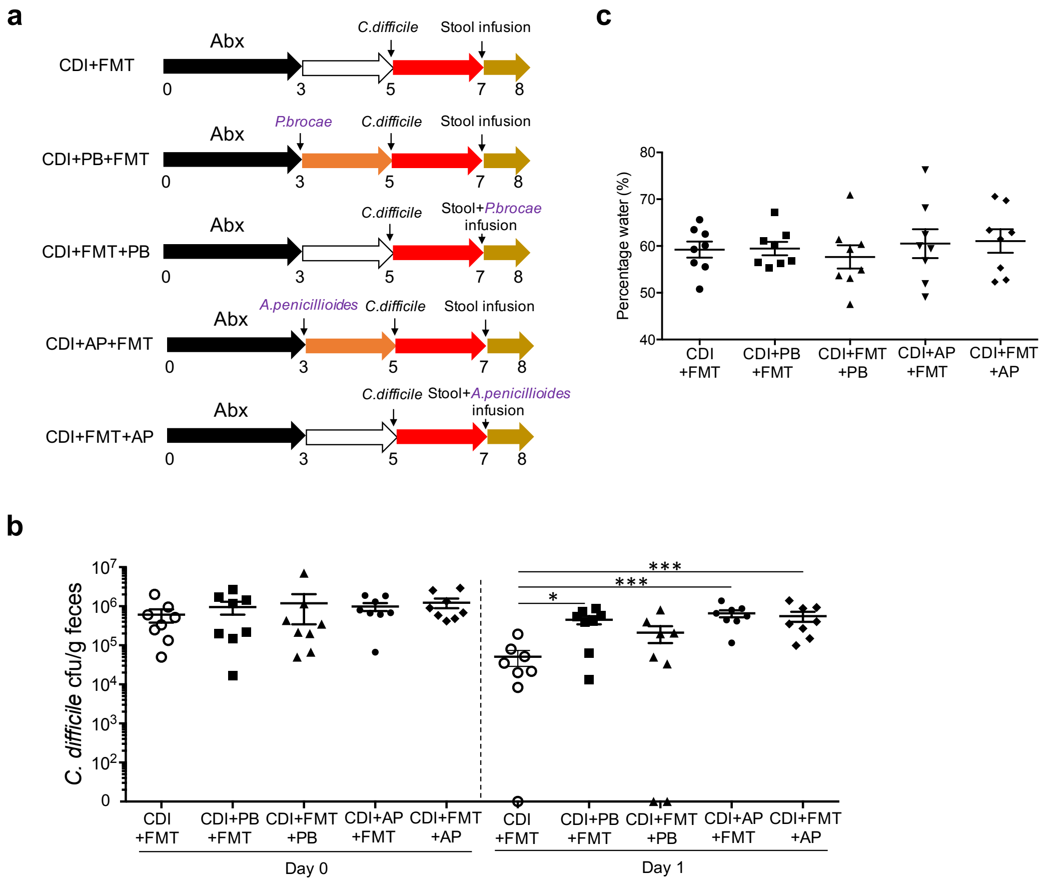

Supplementary Figure 13. Fungi compromise FMT efficacy in eradicating *C.difficile* infection in mice. **a**, Schematic diagram of *P. brocae* (PB) and *A. penicillioides* (AP) administration and stool infusion (FMT) in CDI mice. **b**, Enumeration of *C. difficile* in mouse feces on day 0 before FMT and day 1 post FMT (n=8 mice per group). Statistical significance represents comparisons by unpaired Mann-Whitney test. * *P* <0.05, *** *P* <0.001. **c**, Diarrhea in mice on day 1 after stool infusion. Dot graphs show mean±s.e.m.
