# Supplementary material for: Gut fungal dysbiosis correlates with reduced efficacy of fecal microbiota transplantation in Clostridium difficile infection
Source: Nat Commun. 2018 Sep 10;9:3663. doi: 10.1038/s41467-018-06103-6 (PMC6131390; doi:10.1038/s41467-018-06103-6)
Supplement: Supplementary file 2 — Description of Additional Supplementary Files [file 41467_2018_6103_MOESM2_ESM.pdf]

Description of Additional Supplementary Files

File Name: Supplementary Data 1
Description: Fungal compositions at the species level in the fecal samples of CDI and control subjects.

File Name: Supplementary Data 2
Description: Differentially present fungal taxa between healthy controls and CDI subjects via LefSe analysis at the order, family, genus and species levels.

File Name: Supplementary Data 3
Description: Treatment outcome and metadata of CDI patients.

File Name: Supplementary Data 4
Description: Summary of study subjects and sample metadata.

File Name: Supplementary Data 5
Description: Sequence statistics for stool fungal ITS2 sequencing data.

File Name: Supplementary Data 6
Description: Sequence statistics for stool 16S rRNA sequencing data.
